# End-to-End Sentence-Level Multi-View Lipreading Architecture with Spatial Attention Module Integrated Multiple CNNs and Cascaded Local Self-Attention-CTC

**DOI:** 10.3390/s22093597

**Published:** 2022-05-09

**Authors:** Sanghun Jeon, Mun Sang Kim

**Affiliations:** Center for Healthcare Robotics, Gwangju Institute of Science and Technology (GIST), School of Integrated Technology, Gwangju 61005, Korea; jeon7887@gist.ac.kr

**Keywords:** lipreading, visual speech recognition, multi-view VSR, deep learning, attention mechanism, spatial attention module, convolutional neural network, local self-attention, connectionist temporal classification

## Abstract

Concomitant with the recent advances in deep learning, automatic speech recognition and visual speech recognition (VSR) have received considerable attention. However, although VSR systems must identify speech from both frontal and profile faces in real-world scenarios, most VSR studies have focused solely on frontal face pictures. To address this issue, we propose an end-to-end sentence-level multi-view VSR architecture for faces captured from four different perspectives (frontal, 30°, 45°, and 60°). The encoder uses multiple convolutional neural networks with a spatial attention module to detect minor changes in the mouth patterns of similarly pronounced words, and the decoder uses cascaded local self-attention connectionist temporal classification to collect the details of local contextual information in the immediate vicinity, which results in a substantial performance boost and speedy convergence. To compare the performance of the proposed model for experiments on the OuluVS2 dataset, the dataset was divided into four different perspectives, and the obtained performance improvement was 3.31% (0°), 4.79% (30°), 5.51% (45°), 6.18% (60°), and 4.95% (mean), respectively, compared with the existing state-of-the-art performance, and the average performance improved by 9.1% compared with the baseline. Thus, the suggested design enhances the performance of multi-view VSR and boosts its usefulness in real-world applications.

## 1. Introduction

Hearing and vision, sometimes known as verbal and visual signals, are widely employed in communication. Because audio signals typically include more information than visual signals, various experiments on automatic speech recognition (ASR) have been performed. Consequently, ASR has attained a very high recognition rate without causing significant signal deterioration. Moreover, it has been used in numerous applications. In contrast, visual speech recognition (VSR) recognizes speech content based on the speaker’s lip-movement features in the absence of speech signals, that is, the speech information is inferred from the movement of the lips. In particular, the visual channel receives two-dimensional visual information, which typically contains more redundant information than that contained in the one-dimensional spoken information received via the auditory channel. Overcoming these VSR limitations is challenging.

People with hearing loss frequently communicate using sign language or by reading the movement of the person’s lips. However, sign language has limitations, such as learning and comprehension difficulties, as well as insufficient expression skills. In this regard, VSR can help people with hearing loss interact effectively with others [1,2]. In noisy environments, interference from ambient noise can reduce audio recognition rates. By contrast, the visual information required for VSR does not change; consequently, VSR can increase speech-recognition performance in noisy contexts [3,4]. In particular, owing to the dominance of facial recognition technology in the field of security, including the use of photographs, video playback, and 3D modeling, VSR technology has been subjected to a large number of attacks. In this approach, including lip movement into a security system might improve its reliability [5]. Additionally, conventional speech synthesis can only generate a single voice in the primary domain of visual synthesis, whereas lipreading technology may generate high-resolution speeches of several characters in a video [6]. Furthermore, lip gestures can be employed to increase sign-language identification accuracy or comprehension [7,8].

Recent research has predominantly focused on lipreading from a frontal perspective [9,10,11,12,13,14,15]. This approach contradicts previous findings in the literature showing that human lipreaders prefer non-frontal views [16,17], owing to noticeable lip protrusion and lip rounding at these angles. Therefore, it might be practical to improve frontal-view lipreading abilities using non-frontal lip view information. This information can also be helpful when a frontal view of the mouth, which is the region of interest (ROI), is unavailable. This is true in real-life situations in which the subject’s face is not visible [11,18,19]. In other words, in an audio VSR or VSR system, the speaker is not continually facing the smart device, kiosk, or camera.

Recently, several VSR systems have been proposed [20,21,22,23,24,25,26]. However, most VSR studies focus on frontal facial images because of the shortage of published datasets that include facial images from different angles. These investigations include lipreading studies, in which the emphasis is on frontal, diagonal, and profile images. The OuluVS2 [27] dataset, a publicly accessible multi-view VSR dataset, is typically used as a research corpus for evaluating novel approaches.

Estellers and Thiranin [28] trained a system using both frontal (0°) and profile (90°) faces and performed exploratory research on multi-view lipreading. Their study demonstrated that the frontal perspective exhibited a lower word error rate (WER) than the profile view. Isobe et al. [29] examined the frontal (0°), left profile (90°), and right profile (90°) viewpoints using a multi-angle approach. When the frontal perspective was used instead of the other perspectives, the system performance improved. As a breakthrough sequence-picture encoding approach, Saitoh et al. [21] proposed concatenated frame image encoding (CFI). They developed a framework for a convolutional neural network (CNN) based on CFI and compared two data augmentation methodologies for CFI.

Bauman et al. [16] observed that AI lipreaders perform better when human faces are slightly inclined because of lip protrusion and rounding. They used the active appearance model (AAM) to extract features from five distinct angles. Using a regression technique in feature space to assess lipreading on both view-dependent and view-independent systems, they reported that the view-dependent system outperformed benchmark models in all tests, receiving a perfect score of 30. Aiming at blending diverse views, Zimmermann et al. [22] coupled principal component analysis-based convolutional networks with long short-term memory (LSTM), a deep learning model, a conventional voice recognition model, hidden Markov models, and Gaussian mixture models. They found that a 30° face inclination produced the best effects. Anina et al. [27] recorded the best accuracy at 60°. Lipreading with a profile view produces lower WERs than lipreading with a frontal viewpoint, according to Kumar et al. [20].

Deep learning has also been used to blend multiple view angles and edit photographs. In particular, Komai et al. [30] implemented AAMs to transform frontal faces viewed from various angles. Their results suggested that identification accuracy increased even when the face orientation was rotated roughly 30° from the frontal perspective. The “View2View” system developed by Koumparoulis and Potamianos [23] relies on a CNN-based encoder–decoder paradigm. The technique converts non-frontal mouth photographs into frontal mouth images. Their view-mapping method for VSR and audio-visual speech recognition (AVSR) was reported to be successful.

By synthesizing virtual frontal views from non-frontal images, Estellers et al. [28] devised a position normalization technique and accomplished multi-view speech recognition. Petridis et al. [24] proposed a multi-view bidirectional LSTM-based lipreading model. The proposed approach considers data directly from pixels while simultaneously performing VSR from various perspectives. They discovered that combining the frontal and profile images boosted the accuracy when compared to using only the frontal view. Zimmermann et al. [25] implemented a PCA-based CNN, LSTM network, and GMM–HMM model to extract features in a decision fusion-based lipreading model. They reported that the decision fusion was effective because Viterbi pathways were included. In addition, to perform multi-angle lipreading, Sahrawat et al. [26] employed view-temporal attention to expand a hybrid attention-based connectionist temporal classification (CTC) system. Finally, Lee et al. [31] trained a CNN–LSTM model from beginning to end.

Evidently, numerous studies have been conducted based on deep learning. However, fewer studies have been conducted on multi-view lipreading than existing speech recognition and front lipreading studies.

Therefore, considering the above-mentioned limitations, we propose a multi-view VSR architecture that supports VSR when both frontal and non-frontal lip pictures are identified. In particular, for non-frontal views, we developed an end-to-end sentence-level multi-view lipreading neural-network architecture that outperforms traditional and current deep learning VSR systems. Convolutional, recurrent, and transcriptional layers were sequentially applied to develop the multi-view VSR architecture.

The remainder of this paper is structured as follows: Section 2 delves into the details on the proposed architecture, Section 3 discusses the experiments, and Section 4 discusses the results. Finally, Section 5 provides the concluding remarks of this study.

## 2. Proposed Architecture

**Figure 1:**
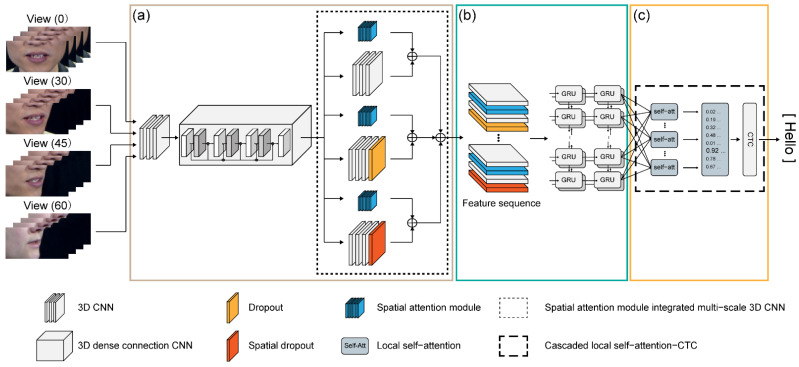
Block diagram of the proposed multi-view lipreading architecture; (**a**) convolutional layer; (**b**) recurrent layer; and (**c**) transcription layer.

In this section, we propose a novel feature-extraction approach. In particular, the proposed architecture is divided into three layers (convolutional layer, recurrent layer, transcription layer) based on an end-to-end neural network with four different perspective inputs, as shown in Figure 1. The three layers are compared against various modules for their performance evaluation. In the convolutional layer, based on the visual extraction module proposed in a previous study [32], the model was modified to improve the feature extraction performance and convergence speed. To compare the modules of the proposed architecture, three current equivalent designs were implemented: multi-scale 3D CNN, spatial attention module (SAM), and integrated multi-scale 3D CNN (Figure 1a). In addition, the recurrent layer was compared as a sequence-processing module with other modules, such as residual neural network (RNN), LSTM, gated recurrent unit (GRU), Bi-LSTM, and Bi-GRU (Figure 1b). The transcription layer was compared as a process for decoding the output features with other components, such as standard CTC, global self-attention-CTC, and local self-attention-CTC (Figure 1c).

### 2.1. Convolutional Layer

To encode visual information from the extracted lips, all input-image sequences were loaded into a spatiotemporal CNN. We extracted spatiotemporal information from an input image composed of numerous continuous frames using a three-dimensional convolutional layer with 64 kernels; 3 × 5 × 5, (1, 2, 2), and (1, 2, 2) are the sizes, strides, and pads, respectively. To minimize the transformation of internal variables, we used a batch normalization (BN) layer and a rectified linear unit (ReLU) layer to accelerate the training process. Subsequently, a max-pooling 3D layer was used to decrease the spatial size of the 3D feature maps. Thus, the output form was observed utilizing 40 × 50 × 25 × 64 tensors with an input sequence of 40 × 100 × 50 × 3 frames.

A densely linked connection contains several connections. In this regard, CNN connects numerous layers of a connection, allowing for efficient feature usage, decreased gradient disappearance, and increased network depth. The input-feature volumes are reduced by the bottleneck layer, which comes before the convolutional layer. The multichannel feature volumes are merged using the bottleneck layer approach. The second layer is applied to only a fraction of the volume of the previous features because the prior features remain visible. Additionally, transition layers are utilized to increase the model’s compactness, with the hyperparameter theta controlling the degree of compression. A bottleneck layer, transition layer, and slower growth rate are used to create a tight network. This strategy saves computing power while minimizing model parameters and preventing overfitting.

**Figure 2:**
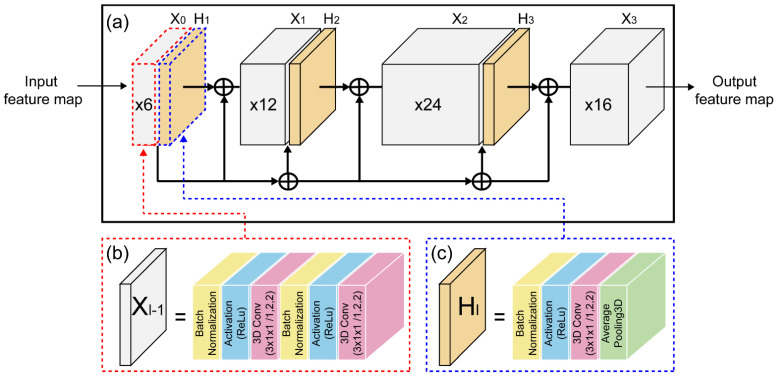
Details of 3D dense connection CNN architecture: (**a**) dense connection CNN; (**b**) dense block layer structure; and (**c**) transition layer structure.

Dense connection CNN is an architecture that focuses on making deep learning networks go even deeper, while simultaneously making them more efficient to train by using shorter connections between the layers (Figure 2). Figure 2a displays a CNN, where each layer is connected to all of the other layers that are deeper in the network, and it consists of two important blocks other than the basic convolutional and pooling layers, that is, the dense blocks and the transition layers. Dense block (1) was built using the following layers in order: BN, ReLU, 3D convolutional, BN, ReLU, and 3D convolutional layers (see Figure 2b). Dense blocks (2), (3), and (4) have the same structure as dense block (1). The transition layer is depicted in Figure 2c, which comprises a BN layer, ReLU layer, three 3D convolutional layers, and two 2D pooling layers.

Different CNN models have yielded outstanding results in picture classification tasks. One such example is feature aggregation using numerous CNNs, which allows the extraction of diverse spatial and temporal information by creating separate structures and depths [33]. Several convolutional layers with varying degrees of abstraction can be extracted during the multi-scale 3D CNN training phase. This training technique can also produce a range of features with various depths and filter sizes. Some of the essential characteristics lost in the layered design can be selected using this strategy, resulting in a more feature-rich final product.

The attention mechanism can boost the feature representation strength of our interests by telling us “what” and “where” to focus our attention. Attention weighting is used in computer vision to boost the feature representation capacity by emphasizing relevant characteristics and limiting inconsequential characteristics. Moreover, attention can be regarded as a strategy for allocating a finite computational force to more informative areas [34,35,36]. Hu et al. [37] proposed the “Squeeze-and-Excitation” module to describe the channel-wise correlation of convolutional features without considering the spatial information. The convolutional block attention module [38] empirically demonstrated that both max-pooling and average-pooling operations contribute to the attention mechanism. Additionally, the inter-spatial interactions feature may be utilized to produce a map of spatial attention. Spatial attention, in contrast to channel attention, focuses on the locations of informative sections and serves as a supplement to channel attention. As a result, the weights associated with attention are distributed over two separate dimensions in this model: channel and space.

**Figure 3:**
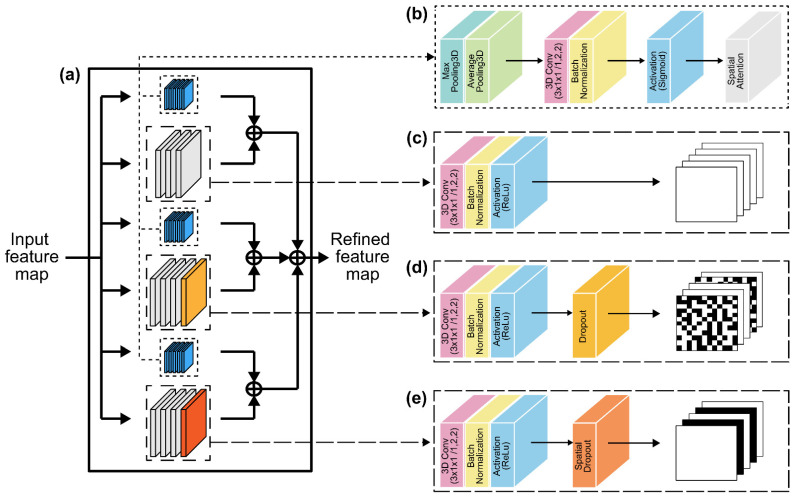
Details of the spatial attention module-integrated MLFF 3D CNN: (**a**) Block diagram of the proposed module; (**b**) spatial attention module; (**c**) first module’s architecture; (**d**) second module’s architecture with dropout layer; and (**e**) third module’s architecture with spatial dropout layer.

The model initially executes average-pooling and max-pooling operations along the channel axis before concatenating them to build an efficient feature descriptor to compute spatial attention. To construct a spatial attention map $M_{s}\left(F\right)\in {ℛ}^{H\times W}$, a convolutional layer is applied to the concatenated feature descriptor. Subsequently, two pooling processes are used to aggregate the channel information of a feature map, resulting in two 3D maps: $F_{\mathrm{avg}}^{s}\in {\mathbb{R}}^{H\times W}$ and $F_{\max}^{s}\in {\mathbb{R}}^{H\times W}$, each representing the average- and max-pooled features over the channel. A 3D spatial attention map is created by concatenating and convolving them with a conventional convolutional layer. In brief, spatial attention is calculated using the following formula:

(1) $$M_{s}\left(F\right)=\sigma (f^{7\times 7}\left(\left[\mathrm{AvgPool}\left(F\right);\mathrm{MaxPool}\left(F\right)\right]\right)),$$

(2) $$M_{s}\left(F\right)=\sigma (f^{7\times 7}\left(\left[{\;F}_{\mathrm{avg}}^{s};F_{\max}^{s}\right]\right)),$$

where σ denotes the sigmoid function, and $f^{7\times 7}$ represents a convolution operation with a filter size of 7 × 7 (Figure 3b).

Because several existing studies implement learning approaches based on sentence front-view datasets [32,39,40,41], it is difficult to expect high accuracy using the same model for multiple viewpoints. Therefore, we propose an SAM-integrated-MLFF 3D CNN, which is a network module focusing on spatial attention with different neighborhoods in the feature maps (Figure 3a). The first module (Figure 3c) comprises a 3D convolutional layer on a 3D dense connection convolutional layer output feature with 32 kernels, followed by a BN layer and a ReLU layer. The second module (Figure 3d) is structured similarly to the benchmark dataset, with a 3D convolutional layer with 64 kernels, followed by a dropout layer to prevent overfitting. By inhibiting the formation of highly correlated activations, the dropout layer enhances and generalizes the performance by avoiding overtraining and overfitting [42].

The third module, which contains a 3D convolutional layer with 96 kernels, is similar to the second module, except for the absence of a dropout layer (Figure 3e). In particular, this method drops the entire feature map. Moreover, in contrast to the traditional dropout method, which removes pixels at random, this method employs CNN models with substantial spatial correlation to improve image classification [43]. Consequently, we employed a spatial dropout layer to extract lips, teeth, and tongue morphologies, which have strong spatial connectivity and contain few movements. Each SAM multi-scale 3D CNN module consists of 3D average-pooling, 3D max-pooling, and 3D convolutional layers, with 32, 64, and 96 3D kernel operations, respectively, along the channel axis and a concatenated BN layer (Figure 3b). Therefore, the output of each multi-scale 3D CNN and SAM is merged and concatenated. As a result, SAM exploits the inter-spatial interaction of the characteristics to better select and focus on the most identifiable and helpful portions of an input picture [38].

### 2.2. Recurrent Layer

Traditional recurrent neural networks (RNNs), LSTM, and GRU are examples of previously implemented RNN algorithms. Owing to the gradient vanishing issue, a typical RNN has difficulties in learning long-range dependent input and output data, owing to the backpropagation technique’s inability to perform adequately with an increase in input data. To overcome this issue, Hochreiter and Schmidhuber [44] created the LSTM network, which is currently widely used in time-series-data processing [45,46,47]. By efficiently overcoming the gradient vanishing issue through effective learning, LSTM and GRU achieve higher levels of validation and prediction accuracy than traditional RNNs, particularly for long-range dependent input and output data [45,47].

A GRU is an RNN that, through multiple stages, learns to manage and transmit information flow [48]. GRUs are constructed using LSTM units that can decide which data to retain and discard. While the 3D CNN only gathers data at the viseme level, GRUs can differentiate across greater temporal contexts, which is crucial for resolving ambiguity. GRU, which consists of an update gate and a reset gate, can also be used to address the gradient vanishing issue.

A two-layer bidirectional GRU is implemented in the proposed architecture, providing a faster convergence speed than a sequence processing module. The two-layer bidirectional GRU is used to transfer information both ways to two distinct neural network topologies coupled to the same output layer, enabling both networks to acquire substantial knowledge of the input. The SAM-integrated-multi-scale 3D CNN provides the input to the two-layer bidirectional GRU layer. For instance, to obtain an output containing 40 × 512 tensors, we submitted a bidirectional GRU 40 × 3 × 1 × 384 frame sequence into the merging layer.

### 2.3. Transcription Layer

Assael et al. [18] used “LipNet” (their neural network, which had outperformed experienced human lip readers) to train a network of end-to-end deep neurons on a benchmark dataset, using the effective CTC loss function [49] for acoustic-based speech recognition. The CTC loss function parameterizes the distribution of the label token sequence without having to align the input sequence; it is conditionally independent of the surrounding distribution generated at each time step. Therefore, the CTC model is a decoding method that uses a beam search technique to detect the temporal dependence of labels.

It is worth noting that the CTC loss function assumes conditional independence of independent labels (i.e., individual character symbols). Each output unit corresponds to the probability of seeing one label at a time. As a result, although CTC is built on RNNs, it is primarily concerned with local data (nearby frames) [50]. While this strategy is effective for forecasting acoustic phonemes, it is not effective for predicting visemes, which require additional background information to discern tiny variations.

**Figure 4:**
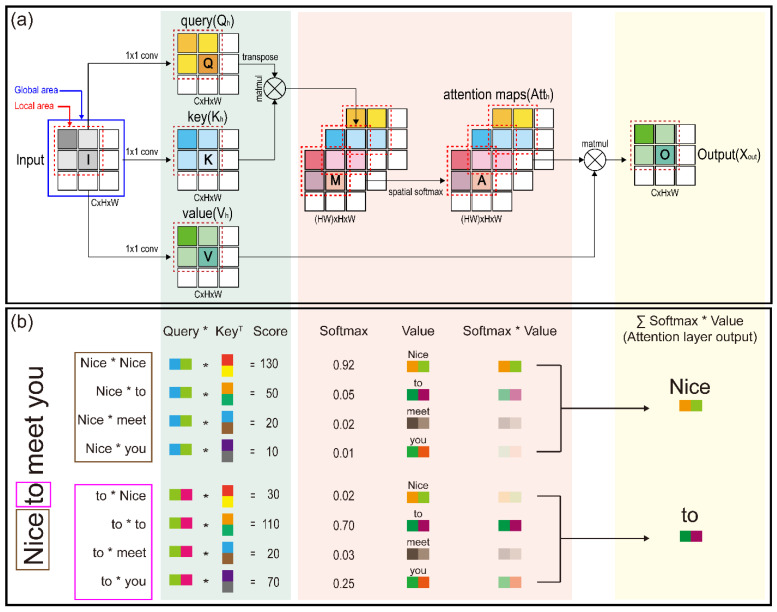
(**a**) Details regarding the global and local self-attention process: the blue line square encloses the global area, and red dotted line square encloses the local area; and (**b**) self-attention mechanism processing process presented for the sentence “Nice to meet you”. (* for dot product).

Figure 4 illustrates that the self-attention mechanism [36,51] is a technique to better encode the word at the target location by looking at the word at another location and taking hints from each word in the input full-sequence sentence. Figure 4a depicts the processing process of the self-attention mechanism, with the global area enclosed by a blue-line square and the local area by a red dotted line. Furthermore, Figure 4b shows an example of the mechanism processing process presented in Figure 4a for the sentence “Nice to meet you”. The multi-head self-attention modules that transformers are known for constitute their distinguishing feature [36]. Given an input $X\in {\mathbb{R}}^{T\times n}$, where T is the number of time steps and n is the hidden state dimension, a set comprising query, key, and value matrices is generated using the weight matrices $W_{h}^{Q}$, $W_{h}^{K}$, and $W_{h}^{V}\in {\mathbb{R}}^{n\times d_{k}}$, respectively, where $d_{k}$ is the dimension of the heads of the attention module. There is one embedding per head, denoted by the subscript h.

(3) $$Q_{h}={\mathrm{XW}}_{h}^{Q},$$

(4) $$K_{h}={\mathrm{XW}}_{h}^{K},$$

(5) $$V_{h}={\mathrm{XW}}_{h}^{V}.$$

The keys and queries are multiplied to obtain a T × T attention matrix A. This matrix encodes the relative relevance of each time step, that is, how much attention each time step receives, by assigning a scalar to each pair of time steps. A SoftMax function with temperature $\sqrt{d_{k}}$ is applied to convert this into a normalized distribution. The value matrix is subsequently multiplied by the normalized attention matrix. Consequently, each time step has a linear combination of value embeddings, with the most significant embedding receiving the largest weights as follows:

(6) $${\mathrm{Att}}_{h}=\mathrm{Softmax}\left(\frac{Q_{h}K_{h}^{T}}{\sqrt{d_{k}}\;}\right)V_{h}.$$

The heads are then concatenated and transformed back to the original dimension *n* using the weight matrix $W^{\mathrm{out}}\in {\mathbb{R}}^{d_{k}\cdot n_{h}\times n}$, where $n_{h}$ is the number of heads. Moreover, a residual connection connecting the output to the input is added as follows:

(7) $$X^{\mathrm{out}}={\mathrm{Concat}}_{h}\left({\mathrm{Att}}_{h}\right)W^{\mathrm{out}}+X.$$

Subsequently, each time step is standardized via layer normalization. For time step t, the overall mean of the feature dimension is subtracted from the input, which is then divided by the standard deviation. This is rescaled and shifted by the learnable parameters α and β as follows:

(8) $$X_{t}^{\mathrm{norm}}=\frac{X_{t}^{\mathrm{out}}-\mu_{t}}{\sigma_{t}}\cdot \alpha +\beta ,$$

where

(9) $$\mu_{t}=\frac{1}{n}\underset{i}{\sum }X_{\mathrm{ti}}^{\mathrm{out}},$$

(10) $$\sigma_{t}=\sqrt{\frac{1}{n}{\left(X_{\mathrm{ti}}^{\mathrm{out}}-\mu_{t}\right)}^{2}}.$$

Next, a feedforward neural network is applied in a time-step-wise manner. This part typically consists of two fully connected layers parameterized by weight matrices $W_{1}\in {\mathbb{R}}^{n\times \phi n}$, $W_{2}\in {\mathbb{R}}^{\phi n\times n}$; bias vectors $b_{1}\in {\mathbb{R}}^{\phi n}$, $b_{2}\in {\mathbb{R}}^{n}$; and a residual connection as follows:

(11) $$f\left(X_{t}^{\mathrm{nrom}}W_{1}+b_{1}\right)W_{2}+b_{2}+X_{t}^{\mathrm{nrom}},$$

where f (·) is an element-wise activation function, such as a ReLU or Gaussian error linear unit. Here, ϕ is a scaling factor for the inner dimensions of the feedforward module. Finally, another layer normalization is applied.

The encoder, decoder, and feedforward contexts were employed to accelerate translation and offer the most current translation findings, sentiment analysis, and other additional operations. The success of self-attention in these tasks motivated the first study on self-attention in speech recognition [52]. As a result, an attention-based encoder–decoder paradigm was devised. Although self-attention was first employed for machine translation, its versatility enabled it to be utilized for voice recognition as well [53,54,55,56]. Attention-based encoder–decoder models rapidly learn the mapping between the auditory frame and the letter sequence. These models generate a label at each output time step based on the input and target label histories. Despite not requiring an external language model, the attention model has a lower character error rate (CER) than CTC. However, the model performs poorly in real-world conditions for various voice recognition tasks, owing to the ease with which noise and other variables may impair the expected alignment in the attention mechanism. Additionally, learning the model from start is difficult, owing to the misalignment of extended input sequences [57,58].

This study used cascaded local self-attention CTC training criteria to improve performance and accelerate learning for the above-mentioned difficulties. When scaling to larger sequences, transformers scale quadratically in the input length. This problem is solved using a unique speech enhancement transformer model based on local attention [59,60]. Local attention is especially well suited for speech augmentation because the predictions do not require long-range correlations, as in natural language processing. Moreover, sufficient information is frequently stored within a few seconds of the target period. Local attention is naturally interwoven with this demand.

The above approach results in huge advances in speech augmentation, where typical sample lengths can involve up to hundreds of thousands of tokens or hours of speech. This small focus incurs only a fraction of the processing and memory overhead associated with attention throughout the entire feature. The windowed technique also allows a more compact packing of padded features in mini-batches, thereby saving costs. Consequently, this module acquires detailed local contextual information from the surrounding area. As the foundational model, we employed cascaded local self-attention with a context size of 12.

## 3. Experimental Evaluation

### 3.1. Dataset

In this study, the proposed architecture was evaluated on the OuluVS2 [27] dataset. This dataset comprises 52 speakers making three types of utterances (Digits, Phrases, and TIMIT), three times each (except TIMIT), simultaneously recorded from five distinct viewpoints (0°, 30°, 45°, 60°, and 90°) for a total of 780 samples per utterance. There are ten classes in total: “Please excuse me”, “Goodbye”, “Hello”, “How are you”, “Nice to meet you”, “See you”, “I am sorry”, “Thank you”, “Have a nice time”, and “You are welcome”. The impact of various mouth ROIs was evaluated by processing the lips from scratch rather than from existing data, and the 90° data were omitted from the experiment because the lips could not be recognized during the extraction process. For the recognition task, we used the Phrase dataset in this investigation. In particular, we utilized the same data split as in other previous studies [21,22,31], to provide a fair comparison. Twelve speakers were used for testing (s06, s08, s09, s15, s26, s30, s34, s43, s44, s49, s51, and s52; 10 men and 2 women) and 40 for training from the database (s06, s08, s09, s15, s26, s30, s34, s43, s44, s49, and s51). Note that s29 is not included in the list.

### 3.2. Data Preprocessing and Augmentation

A DLib face detector [61] was used in the data-preparation step to recognize the targeted face and mouth. A HoG feature-based linear classifier [33] was used in the detector. The diagonal edges’ (x, y) coordinates were obtained and used to build a bounding box around the mouth. As a result, the iBug program was used to forecast facial landmarks [62], considering 68 landmarks and an online Kalman filter. This method is widely used to extract the lip points that match with those in the training dataset by reading lip motions. These algorithms were utilized to extract a mouth region from each frame, and to perform an affine transformation to equalize the RGB channels throughout the training set, resulting in a mean and variance of zero. Moreover, we employed a data augmentation approach for training data to avoid overfitting [18]. The training process considered both standard and horizontally mirrored picture sequences. The degradation rate for these occurrences was 0.925. Finally, to avoid variance, we identified the movement speed and repeated each frame with a probability of 0.05. All models were trained and evaluated on the OuluVS2 dataset, using identical preprocessing and augmentation methods.

### 3.3. Implementation

To evaluate the performance of the CTC decoder, all models used Keras, based on TensorFlow backend on Linux Ubuntu; the computer had an Intel^®^ Core™ i7-7700K processor, along with 64GB RAM and an NVIDIA GeForce RTX 2080-Ti GPU. The hyperparameters specified in Table 1 are the values for each layer of the proposed model. The network parameters—other than the initialized GRU matrix and hyperparameters—were initialized for all models. To perform the optimization of models, adaptive moment estimation (Adam) [63], stochastic gradient descent (SGD) [64], RMSprop [65], AdaMax, and Nadam [64] optimizers were used in mini-batches of sizes 8 and 0.0001, trained at the learning rate. The proposed model was trained in a multi-scale 3D CNN with SAM; channel-wise dropped pixels and spatial dropout for the dropped channel were used, and the proposed model contained the baseline model, trained on the dataset until it was overfitted. The moving average strategy was used to smooth it down for better viewing. Regarding the accuracy of the proposed model, the genuine value was represented by the shadow part of the image, while the curve represented the smoothed value. We selected a smaller batch size of 75 images owing to the computer’s restricted capabilities, causing the real value fluctuation to be uneven. Smoothing was performed to alleviate this problem and to make the curves comprehensible.

### 3.4. Performance Evaluation Metrics

We used standard automated speech-recognition assessment criteria as the evaluation metrics. The learning loss of each model was calculated to determine its learning status during the training operation. Furthermore, we compared each model’s performance and computational efficiency by examining its parameters, epoch period, and CER.

For the misclassification analysis, it is necessary to compare the original text and the predicted text. The five variables used in the equation are the characters (C), the total number of ground truth characters (N), the false predicted characters (S), the non-selected characters (I), and the number of deleted characters (D). CTC beam search is performed for maximum probability prediction, and the CER equation is as follows:

(12) $$\mathrm{CER}\;\left(\%\right)=\left(\frac{C_{S}+C_{D}+C_{I}}{C_{N}}\right)\times 100,$$

We compared the CER for parameter count and computational efficiency during the study period. The results are presented using a confusion matrix.

## 4. Results

### 4.1. Learning Loss and Convergence Rate

**Figure 5:**
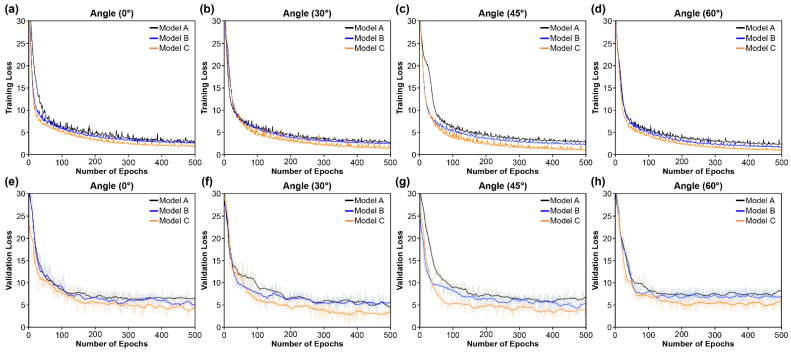
Training and validation loss comparing convergence speed of convolutional layers (Models A, B, and C): (**a**–**d**) Training loss at (**a**) 0°; (**b**) 30°; (**c**) 45°; and (**d**) 60°; (**e**–**h**) Validation loss at (**e**) 0°; (**f**) 30°; (**g**) 45°; and (**h**) 60°.

**Figure 6:**
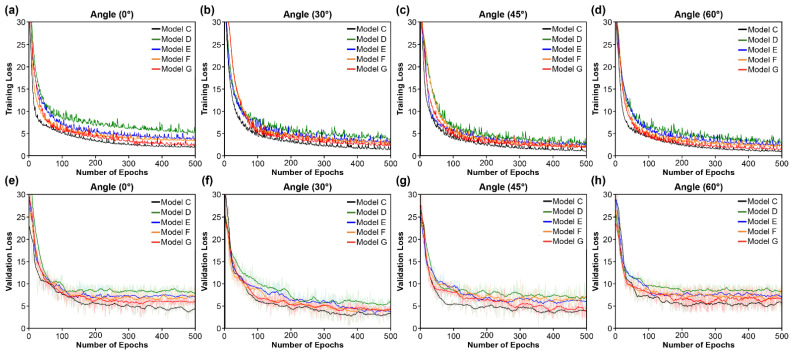
Training and validation loss comparing convergence speed of recurrent layers (Models C, D, E, F, and G). (**a**–**d**) Training loss at (**a**) 0°; (**b**) 30°; (**c**) 45°; (**d**) 60°. (**e**–**h**) Validation loss at (**e**) 0°; (**f**) 30°; (**g**) 45°; (**h**) 60°.

**Figure 7:**
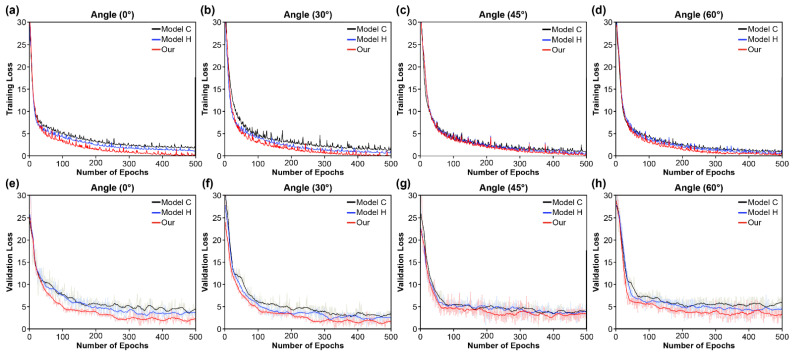
Training and validation loss comparing convergence speed of transcription layers (Model C, Model H, and the proposed model). (**a**–**d**) Training loss at (**a**) 0°; (**b**) 30°; (**c**) 45°; (**d**) 60°; (**e**–**h**) Validation loss at (**e**) 0°; (**f**) 30°; (**g**) 45°; (**h**) 60°.

Figure 5, Figure 6 and Figure 7 compare the learning loss and convergence speed rates for the convolutional, recurrent, and transcription layers, respectively. Figure 5 shows the learning loss (training and validation) on the OuluVS2 dataset for the convergence rates of the three types of CNNs in the convolutional layer. The three models have different visual feature extraction modules at the front end, and the same recurrent and transcription layers at the back end. Model A consists of a densely connected 3D CNN, Model B combines the multi-scale 3D structure following Model A, and Model C is configured by combining a SAM with Model B. In addition, Figure 5 shows that the training and validation losses of all three models are similar from all four angles. However, the gap between the training and validation losses was the highest in Model A, and its degree of overfitting was higher than those of the other models. Furthermore, although Model C increased the number of parameters by 30 M compared to Model A, it exhibited lower overfitting results (the smallest among all models) (Figure 5). This is because Model A comprised a model with outstanding performance based on the DenseNet-121 [66] structure, thereby minimizing the number of model parameters, successfully suppressing overfitting, and saving computation. However, the combination of multi-scale 3D CNN (Model B) and SAM (Model C) yielded improved results because this combination identified better by focusing on the most distinguishable and beneficial areas of the input image. Therefore, the learning and convergence speeds of Model C were high, and the gap was small. These findings indicate that the proposed model had the smallest difference between the training and validation losses, preventing overfitting on the OuluVS2 dataset.

Figure 6 shows the learning loss (training and validation) on the OuluVS2 dataset for the convergence rates of the four types of RNN in the recurrent layer. The convolutional and transcription layers had the same structure, and only the configuration of the recurrent layers differed. The Bi-GRU exhibited the fastest learning convergence speed and best prediction accuracy, as shown in Figure 6 and Figure 9e–f. In particular, all four RNN unit types outperformed the RNN. The experimental results and prediction accuracy are similar to the findings reported in Section 5 of [44], where LSTM and GRU displayed improved validation accuracy and prediction accuracy compared to traditional RNNs (Table 2), owing to their resistance to the vanishing gradient problem. Compared with LSTM and Bi-LSTM, both GRU and Bi-GRU demonstrated faster convergence and lower losses. The bidirectional models outperformed the unidirectional models on the training set for both GRU and LSTM; they also outperformed their unidirectional counterparts on the validation dataset. Consequently, Bi-GRU exhibited the best overall performance.

The learning loss (training and validation) on the OuluVS2 dataset is shown in Figure 7 for the convergence rates of the proposed model’s three types of CTC loss functions in the transcription layer. The convergence rate for learning was slower than that in the other two situations, when only the basic CTC loss function was used. In particular, as the angle of the detected lip changed, the convergence rate further decreased, while the two cases of cascaded self-attention exhibited similar convergence rate tendencies for all of the angles. The two self-attention modules learned with similar convergence rate tendencies. However, in all of the four results shown in Figure 7, the local self-attention module exhibited a faster convergence rate than the global self-attention modules. First, the principle of the CTC loss function assumes conditional independence for each label, and, since each output unit denotes the probability of seeing a single label at a given moment, it provides a high premium to the nearby local information [50]. Thus, ineffectiveness in predicting visemes is a possible reason for the difference in convergence rates.

The cascaded self-attention CTC module (which generates an output sequence with long-term temporal correlation) increases the speed of convergence, as compared to the CTC decoder (which assumes the input is conditionally independent). The attention approach is used in the CTC decoder’s pre-alignment stage to remove unnecessary paths. The CTC decoder is then used to align the video frames and text labels, thereby allowing the attention mechanism to focus on the video–text pairs in the correct order. As a result, fewer irrelevant samples are created, resulting in the observed speedup. Second, the local self-attention module’s windowed method results in more compact packaging of the padded features in mini-batches, and, hence, further cost reductions. Consequently, this local self-attention requires only a fraction of the computing and memory costs of attention over the entire feature, while providing rich local contextual information in the small region.

### 4.2. Optimization

The update rules of the optimization algorithms are usually defined by the hyperparameters that influence their behavior (e.g., the learning rate). The optimizer’s responsibility is to update the weight parameters prior to reducing the error or loss function, which is the difference between the actual and predicted values. This requires several iterations with varying weights. However, choosing an optimizer for network training can be tricky. Deep learning employs iterative rules to modify or evaluate the data, utilizing numerous aspects and techniques. Therefore, training models as quickly as possible is vital to complete the iterative cycle and, as a result, enhance the prediction accuracy and speed. Consequently, in this part, we study the following optimizers used to train deep learning neural networks: SGD, RMSprop, Adam, Nesterov-accelerated Adam (Nadam), and AdaMax. After validating that AdaDelta and AdaGrad diverged without learning throughout the learning process, we omitted them from the experiments.

SGD realizes one update at a time to avoid duplication, making it significantly faster and easier to learn than other deep learning neural networks [67]. These frequent updates of the method with high variance introduce significant fluctuation in the objective function. This variation allows the parameters to move into new, possibly better, local minima. However, as SGD continues to overshoot, converging to the precise minimum is challenging. The parameters of AdaDelta have varying learning speeds, and the learning process comes to a halt after a certain point. This problem was addressed using the RMSprop method [65]. For each sample in each iteration, RMSprop uses a variable learning rate that is changed according to the results. RMSprop calculates the average of the first-order moments of the gradients and accelerates convergence by ignoring distant previous locations. Moreover, the squares of gradients and the average of the second-order moments are considered by AdaDelta and RMSprop. In the Adam optimizer, the adaptive optimization method is applied. Based on the parameters to be used, this optimizer dynamically modifies the learning rate for each sample in the dataset. Adam is a fast thinker with a limited memory span. Therefore, SGD, AdaDelta, and RMSprop [65] were used to create this algorithm.

Nadam combines Adam and Nesterov momentum. This method was developed similarly to Adam, with the exception that the flat momentum is replaced with the Nesterov momentum. The substitution causes a more considerable increase in performance than that in momentum. [63,68]. Alternatively, AdaMax, an extension of the Adam optimizer, was developed [63]. To update the weight parameters in AdaMax, the infinity norm of the moment is used, instead of the second-order moment estimate. Therefore, the size of the parameter update in AdaMax has a simpler constraint structure than in Adam, and the weight-updating rules are stable.

**Figure 8:**
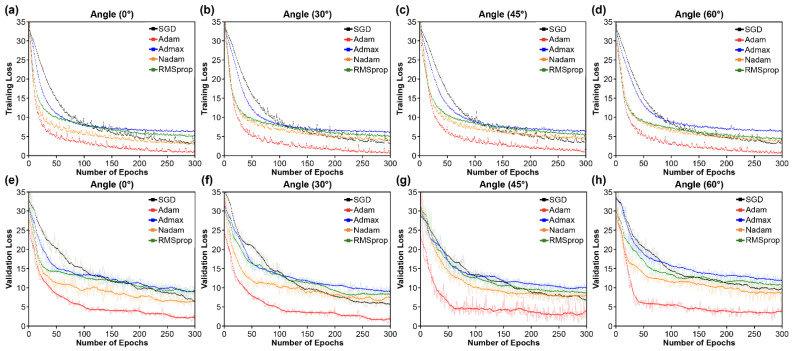
Loss curves comparing various optimizers. (**a**–**d**) Training loss at (**a**) 0°; (**b**) 30°; (**c**) 45°; and (**d**) 60°; (**e**–**h**) validation loss at (**e**) 0°; (**f**) 30°; (**g**) 45°; and (**h**) 60°.

We used the Bi-GRU classifier to compare the training results and determine the most successful optimizer. Figure 8 depicts the loss curves of the optimizers. In particular, Adam performed better among the optimizers at all of the four angles. The Adam optimizer’s loss converged at the quickest pace, implying that it trained the Bi-GRU classifier more successfully than the other algorithms. The results show that Adam was the best optimizer for training the Bi-GRU architecture’s lip-based classification model. Therefore, this approach was employed in further trials in this study to train the Bi-GRU classifier.

### 4.3. Performance and Accuracy

**Figure 9:**
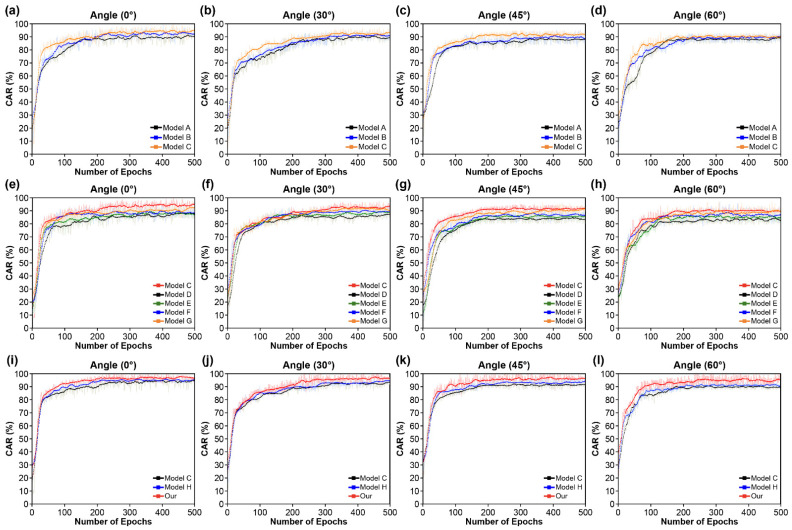
Training steps for character error rate (CER) comparing our proposed model to the baseline and other models: (**a**–**d**) Convolutional layer; (**e**–**h**) recurrent layer; (**i**–**l**) transcription layer.

The results presented in this section correspond to the OuluVS2 dataset phrases. Table 2 and Table 3 show that the proposed model outperformed existing deep learning models by attaining state-of-the-art (SOTA) results: 3.31% (0°), 4.79% (30°), 5.51% (45°), 6.18% (60°), and 4.95% (mean). These results show an improvement over the previous SOTA results in all of the conditions. Figure 9 compares the accuracy results between the models by dividing them into three layers: convolutional layer (Figure 9a–d), recurrent layer (Figure 9e–h), and transcription layer (Figure 9i–l).

In the case of the convolutional layer (Figure 9a–d and Table 2), on average, the performance improved by 3.63% for all of the four angles when MLFF 3D CNN and SAM were combined than when only the DenseNet-121 structure was used. By combining the SAM with MLFF 3D CNN, a 2.46% improvement was observed owing to improved recognition among the inter-spatial relationships of features. This helped to better identify and focus on the most distinguishable and informative areas of the input image.

In the case of the recurrent layer (Figure 9e–h and Table 2), five RNN units (RNN, LSTM, Bi-LSTM, GRU, and Bi-GRU) were compared. For all of the four angles, LSTM and GRU exhibited higher accuracy than the standard RNN. This is because of their robustness against gradient disappearance, which allows them to successfully learn long-range dependent input data. Therefore, the average accuracy of LSTM increased by 1.83% compared to when RNN was used. Similarly, the average accuracy of GRU increased by 4.17%. However, despite its similar performance, Bi-LSTM’s accuracy increased by 2.71% compared to RNN, and Bi-GRU’s accuracy improved by 6.77% when unidirectional models were used, compared to bidirectional models. The bidirectional models also achieved better results on the validation dataset than their unidirectional counterparts. Thus, the best overall performance was achieved using the Bi-GRU.

In the case of the transcription layer (Figure 9i–l and Table 2), we compared the performance by combining the global and local self-attention mechanisms with the basic CTC function in the cascade method. For all of the four angles, the two CTC loss functions exhibited higher performance than the basic CTC loss function. When using the global self-attention method, accuracy improved by 0.95%, while the local self-attention method improved by 5.47%. The performance of the two models is better than that of the CTC loss function because they overcome the disadvantage of assuming a conditionally independent input. Moreover, the performance difference between the two methods exists because the local self-attention module led to a more compact packing of the padded features in mini-batches, resulting in additional savings. Therefore, this local self-attention required a fraction of the compute and memory costs associated with attention over the entire feature and rich local contextual information in the local region. Thus, the proposed model surpasses current models, including the experimental model, in terms of accuracy, which can be attributed to the three layers. The training approach with three layers is illustrated in Figure 9, using the OuluVS2 dataset.

### 4.4. Statistical Analysis and Model Efficiency

**Figure 10:**
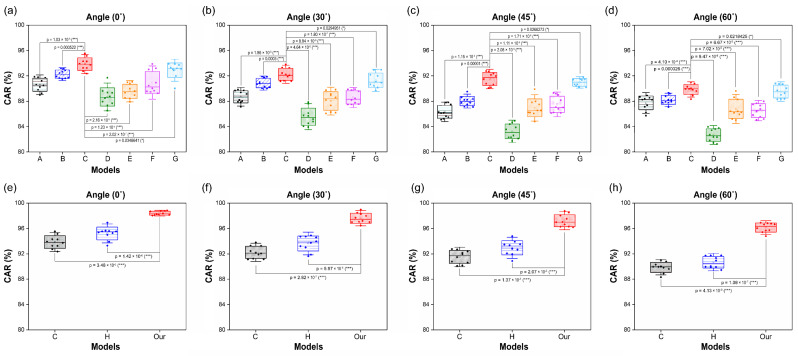
Comparison between different models and the proposed model based on mean accuracy of the last 10 epochs: (**a**–**d**) Convolutional layer and recurrent layer; (**e**–**h**) Transcription layer. Error bars represent standard deviation. Asterisks represent statistical significance-based *t*-tests between each group (* for *p* < 0.05, ** for *p* < 0.01, and *** for *p* < 0.001).

We performed statistical analysis using the standard *t*-test to compare the significance of the combined modules. Models A and B of the convolutional layer were compared, based on Model C (Figure 10a–d), and Models C, D, E, F, and G were compared in the current layer (Figure 10a–d). In addition, in the transcription layer, Models C and H and the proposed model were compared (Figure 10e–h). For all four angles in Figure 10a–d, the proposed model showed that the modules in the convolutional layer have significant differences. That is, the performance increased by combining the MLFF 3D CNN and the SAM with the DenseNet-121 model. In addition, in the recurrent layer, the use of the Bi-GRU classifier (Model C) exhibited the highest performance and significant results compared to the four RNN-type units. However, in the case of Model G, because the unidirectional GRU model was used, there was no significant difference compared to Model C, which is a bidirectional model. Figure 10e–h shows the statistical analysis of the transcription layer. The performance of the two models using the self-attention mechanism in the cascade method was higher and significant than that for learning based on the basic CTC loss function. Consequently, the proposed model exhibited significant performance improvement.

**Figure 11:**
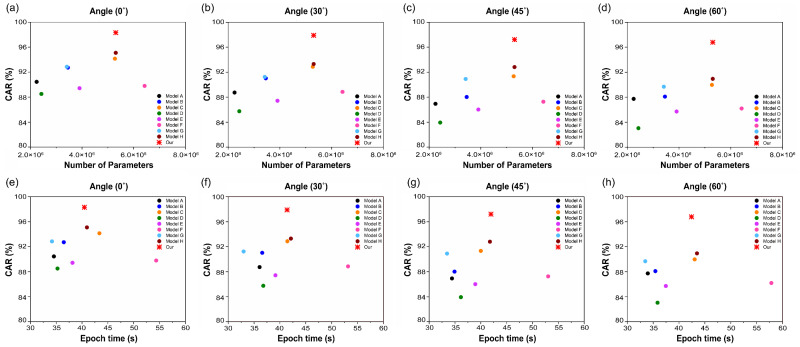
Comparison of character accuracy rate (CAR) between the proposed model and other models according to the (**a**–**d**) number of parameters and (**e**–**h**) average epoch time.

In practical applications, the primary limitations of the VSR systems are their size and computing capacity. We explored the models’ computational efficiency by examining their accuracy over various training settings and epochs. The system’s performance as a function of the number of parameters is shown in Figure 11a–d. Furthermore, Figure 11e–h depict the results of the average epoch–time comparison of the nine models for 500 epochs. As demonstrated in Table 4, each model on the OuluVS2 dataset has a unique set of parameters and epoch time. Compared to Model D, which presented the lowest accuracy among the compared models, the proposed model had a parameter count difference of approximately 29 M. The average accuracy was improved by 12.24%. In comparison to Model F, which had the most parameters, the proposed approach decreased the number of parameters by roughly 11 M, while increasing accuracy by 9.53%. In addition, the difference in learning time compared to Model D, with the smallest number of parameters, differed by 5.54 s on average per epoch, which is not significant. Furthermore, the difference in learning time compared to that of Model F, which has the most parameters, was 13.05 s. Thus, the proposed model is capable of enhancing accuracy and decreasing learning time without considerably increasing the number of parameters.

### 4.5. Confusion Matrix

**Figure 12:**
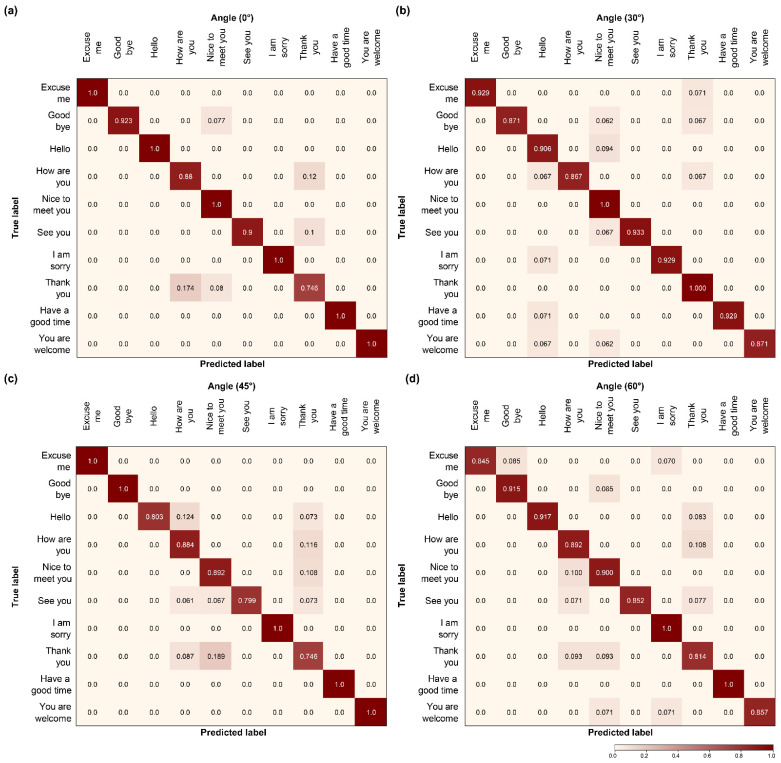
Comparison of confusion matrix models: (**a**–**d**) Model C.

**Figure 13:**
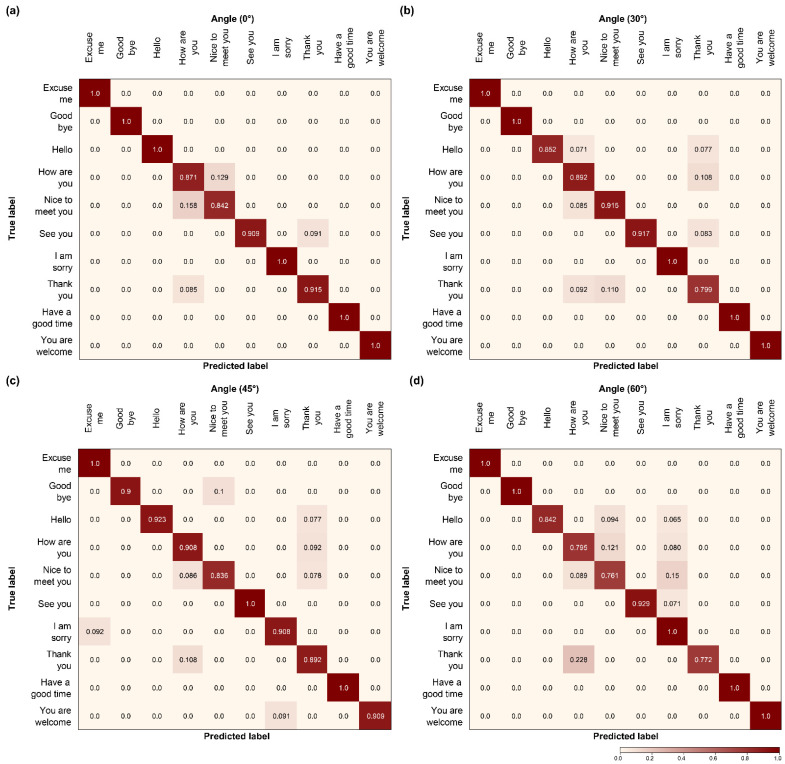
Comparison of confusion matrix models: (**a**–**d**) Model H.

**Figure 14:**
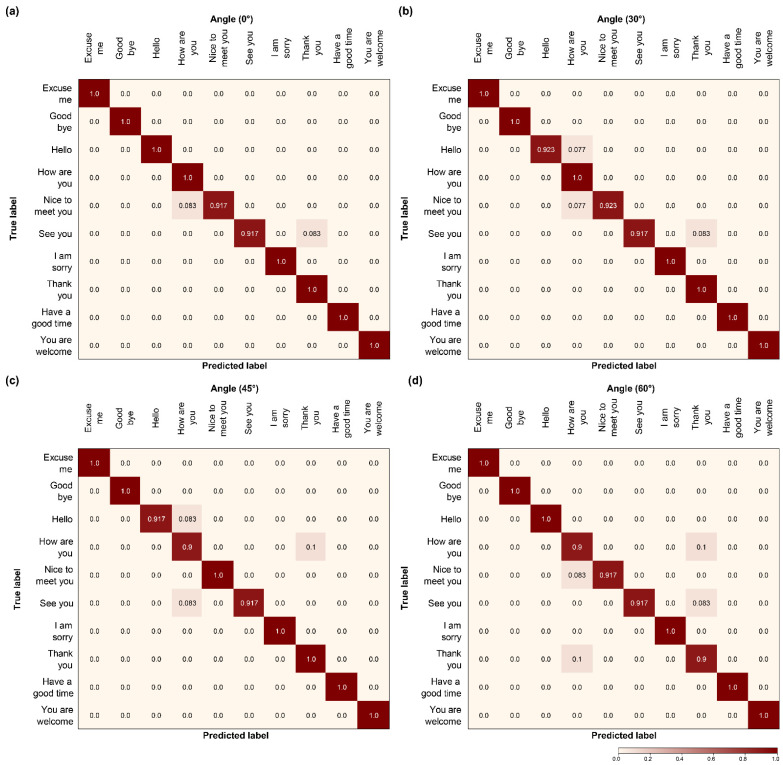
Comparison of confusion matrix models: (**a**–**d**) the proposed model.

We compared the confusion matrices of the two models that exhibited outstanding performance in the three layers with that of the proposed model for the four angles. Specifically, we evaluated Model C (Figure 12), which exhibited the highest accuracy in the convolutional and recurrent layers; Model H (Figure 13), which exhibited excellent performance in the transcription layer; and the proposed model (Figure 14). When comparing the results shown in Figure 12, the proposed model realizes fewer incorrect predictions. In addition, Model C had more erroneous predictions than the other two models for the four angles. The number was particularly high for “Hello”, “Thank you”, and “See you” because they are visually similar from the same viewpoint, furthermore, “Thank you” and “See you” have identical viseme sequences around the beginning and end of the utterance, which explains why these phase pairings have a higher number of false predictions. Because they are visually comparable from the same viewpoint, the three pairs of sentences with the highest error rate are the most demanding and confusing pairings with a high error rate, as indicated by the confusion matrix [13,24,31].

However, when the global self-attention mechanism was combined with the transcription layer, Model H exhibited better overall confusion pair results than Model C in 10 phases. Model H clearly demonstrated that confusion decreased compared to Model C. Despite the decrease in confusion, some pairs show particularly high confusion rates at each angle. As can be observed in Figure 13a, the predictions between “Nice to meet you” and “How are you” were the lowest, and, as shown in Figure 13b,c, were confused with “Nice to meet you” and “How are you” for “Thank you.” In addition, unlike the other three angles, the 60° angle (Figure 13d) showed substantial confusion, wherein “Thank you” and “How are you” exhibited the lowest predictions. Therefore, Model H, similar to Model C, increased the number of confusions, due to the similarity of the visual view as the angle increased. The last pronunciation, such as “you”, showed low predictions within a similar phase.

Unlike the two models, the proposed model yields low confusion at all of the angles using the local self-attention mechanism. In particular, for the 60° angle, both Models C (Figure 12d) and H (Figure 13d) presented high confusion numbers. In contrast, the proposed model (Figure 14d) presented low confusion numbers, similar to other angles. In addition, the confusion between “Hello”, “Thank you”, and “See you” observed in the other two models was reduced, and the predicted value increased. By comparing the confusion matrices, we can easily define which of the models performs better. Thus, we can establish that the proposed model outperformed the others on the OuluVS2 dataset, distinguishing all comparable pronunciations in phase.

## 5. Discussion and Conclusions

Lipreading is difficult to execute because it cannot be purely performed from the frontal perspective. Professional lip readers claim that a non-faceted approach, instead of a front-view, provides more information than a front-view with more pronounced lip protrusions and lip rounding. Consequently, the most significant limitation in using lipreading technology in real-world applications is its performance when reading lips from multiple angles. Therefore, we developed a multi-angle/multi-view VSR architecture that performs VSR by detecting both frontal and non-frontal lip images.

This study provides an end-to-end infrastructure for recording multi-view video surveillance. We obtained an accurate viseme prediction using SAM, multiple CNNs, and cascaded local self-attention-CTC. This is the first time that a 3D CNN, 3D dense connection CNN, and SAM have been combined with a multi-scale 3D CNN to extract lip motion characteristics as encoders. Following the decoder’s Bi-GRU, a transcription layer based on cascaded local self-attention-CTC was used to extract exhaustive local contextual information from the surrounding environment.

The advantages of each level of the proposed architecture can be summarized as follows. The 3D dense connection CNN helps in reducing gradient vanishing and deepening the network (to use features) in an efficient manner. It also helps in reducing model parameters and preventing overfitting, thereby conserving computational resources. Finally, the multi-scale 3D CNN is applied to the two dropout layers, using features at different levels to effectively analyze the motion context in the temporal and spatial domains, with fine motion and high spatial correlation. SAM and multi-scale 3D CNNs are combined and concatenated to provide a single output. Consequently, SAM exploits the inter-spatial interaction of characteristics to better select and focus on the most identifiable and practical portions of an input picture. Moreover, cascaded local self-attention-CTC, following the decoder’s Bi-GRU, requires only a fraction of the computation and memory costs of attention over the entire feature, leading to compact packaging of padded features in mini-batches and significant savings. Hence, this module can be used to acquire detailed local contextual information from the surrounding area.

We compared the outcomes of various deep learning models for predicting the sequence of phrases. The proposed architecture outperformed the others in terms of SOTA CER (Table 2 and Table 3). We also compared the convergence rate, optimization, accuracy, statistical analysis, model efficiency, and confusion of the learning process for the three layers (convolution, recurrent, and transcription). The proposed model exhibited a faster convergence speed and higher accuracy compared to the other models, without a significant difference in the number of parameters and epoch time.

The proposed model attained SOTA performance on the OuluVS2 dataset without requiring external data or even data augmentation. The given mouth ROIs, on the other hand, were appropriately cropped, which may not be the case when employing automated mouth ROI identification techniques. Additionally, it would be interesting to investigate the effect of automated mouth ROI cropping on multi-view lipreading because the accuracy of automatic detectors is known to degrade with non-frontal views. Finally, because the model can be readily expanded to other streams, we expect to incorporate an audio stream to see how well it performs in audio-visual multi-view speech recognition.

Developing a multi-view VSR system that exclusively relies on visual data is crucial. Speech recognition in loud situations, hearing impairment, and biometric identification are some applications for which such a system will be practical. It could also be helpful for people with speech difficulties. However, because speech involves auditory and visual information, it is still challenging to perform ASR simply by using VSR. As a result, we plan to widen our approach in the future to include performance optimization and identification of potential uses for audio and visual data.

## Figures and Tables

**Table 1:** Hyperparameters of the proposed architecture.

Layer	Output Shape	Size/Stride/Pad		Dimension Order
Input Layer	40 × 100 × 50 × 3	-		T × C × H × W
Convolution 3D Layer	40 × 50 × 25 × 64	[3 × 5 × 5]/(1, 2, 2)/(1, 2, 2)		
		[1 × 2 × 2] max pool/(1 × 2 × 2)		
3D Dense Block (1)	40 × 25 × 13 × 96	[3 × 1 × 1] 3D Conv	(×6)	
		[3 × 3 × 3] 3D Conv		
3D Transition Block (1)	40 × 12 × 6 × 6	[3 × 1 × 1] 3D Conv		
		[1 × 2 × 2] average pool/(1 × 2 × 2)		
3D Dense Block (2)	40 × 12 × 6 × 38	[3 × 1 × 1] 3D Conv	(×12)	
		[3 × 3 × 3] 3D Conv		
3D Transition Block (2)	40 × 6 × 3 × 3	[3 × 1 × 1] 3D Conv		
		[1 × 2 × 2] average pool/(1 × 2 × 2)		
3D Dense Block (3)	40 × 6 × 3 × 35	[3 × 1 × 1] 3D Conv	(×24)	
		[3 × 3 × 3] 3D Conv		
3D Transition Block (3)	40 × 3 × 1 × 1	[3 × 1 × 1] 3D Conv		
		[1 × 2 × 2] average pool/(1 × 2 × 2)		
3D Dense Block (4)	40 × 3 × 1 × 33	[3 × 1 × 1] 3D Conv	(×16)	
		[3 × 3 × 3] 3D Conv		
Multi-scale 3D CNN (1)	40 × 3 × 1 × 32	[3 × 5 × 5]/(1, 2, 2)/(1, 2, 2)		
Multi-scale 3D CNN (2)	40 × 3 × 1 × 64	[3 × 5 × 5]/(1, 2, 2)/(1, 2, 2)		
Multi-scale 3D CNN (3)	40 × 3 × 1 × 192	[3 × 5 × 5]/(1, 2, 2)/(1, 2, 2)		
Spatial Attention (1)	40 × 3 × 1 × 32	[1 × 2 × 2] max pool/(1 × 2 × 2)		
		[1 × 2 × 2] average pool/(1 × 2 × 2)		
		[3 × 7 × 7]/(1, 2, 2)/(1, 2, 2)		
Spatial Attention (2)	40 × 3 × 1 × 64	[1 × 2 × 2] max pool/(1 × 2 × 2)		
		[1 × 2 × 2] average pool/(1 × 2 × 2)		
		[3 × 7 × 7]/(1, 2, 2)/(1, 2, 2)		
Spatial Attention (3)	40 × 3 × 1 × 96	[1 × 2 × 2] max pool/(1 × 2 × 2)		
		[1 × 2 × 2] average pool/(1 × 2 × 2)		
		[3 × 7 × 7]/(1, 2, 2)/(1, 2, 2)		
Bidirectional GRU Layer	40 × 512	256		T × F
Bidirectional GRU Layer	40 × 512	256		T × F
Local Self-Attention Layer	40 × 512	15		T × F
Dense Layer	40 × 28	27 + blank		T × F
SoftMax Layer	40 × 28			T × V

**Table 2:** Performance of the proposed model compared to various models on the OuluVS2 dataset.

Model	Method	Top 10 Accuracy (%)				
		0°	30°	45°	60°	Mean
A *	3D dense connection CNN + Bi-GRU + CTC	90.44	88.73	86.93	87.72	88.45
B *	3D dense connection CNN + Multi-scale 3D CNN + Bi-GRU + CTC	92.72	91.02	88.02	88.09	89.62
C *	3D dense connection CNN + Multi-scale 3D CNN + SAM + Bi-GRU + CTC	94.14	92.86	91.34	89.97	92.08
D *	3D dense connection CNN + Multi-scale 3D CNN + SAM + RNN + CTC	88.51	85.74	83.93	83.04	85.31
E *	3D dense connection CNN + Multi-scale 3D CNN + SAM + LSTM + CTC	89.42	87.42	86.01	85.71	87.14
F *	3D dense connection CNN + Multi-scale 3D CNN + SAM + Bi-LSTM + CTC	89.78	88.84	87.26	86.18	88.02
G *	3D dense connection CNN + Multi-scale 3D CNN + SAM + GRU + CTC	92.85	91.23	90.91	89.67	91.14
H *	3D dense connection CNN + Multi-scale 3D CNN + SAM + Bi-GRU + Global self-attention + CTC	95.08	93.29	92.81	90.93	93.03
Our *	3D dense connection CNN + Multi-scale 3D CNN + SAM + Bi-GRU + Local self-attention + CTC	98.31	97.89	97.21	96.78	97.55

* Model trained with data augmentation.

**Table 3:** Performance of existing models on the OuluVS2 dataset.

Year	Model	0° (%)	30° (%)	45° (%)	60° (%)	Mean (%)
2014	RAW-PLVM [69]	73.00	75.00	76.00	75.00	74.75
2016	CNN * [21]	85.60	82.50	82.50	83.30	83.48
	CNN + LSTM [31]	81.10	80.00	76.90	69.20	76.80
	CNN + LSTM, Cross-view Training [31]	82.80	81.10	85.00	83.60	83.13
	PCA Network + LSTM + GMM–HMM [22]	74.10	76.80	68.70	63.70	70.83
	CNN pretrained on BBC dataset * [52]	93.20	-	-	-	-
	CNN pretrained on BBC dataset + LSTM * [70]	94.10	-	-	-	-
2017	End-to-End Encoder + BLSTM [24]	94.70	89.70	90.60	87.50	90.63
	Multi-view SyncNet + LSTM * [71]	91.10	90.80	90.00	90.00	90.48
	End-to-End Encoder + BLSTM [13]	84.50	-	-	-	84.50
	End-to-End Encoder + BLSTM [72]	91.80	87.30	88.80	86.40	88.58
2018	CNN + Bi-LSTM [73]	90.30	84.70	90.60	88.60	88.55
	CNN + Bi-LSTM [73]	95.00	93.10	91.70	90.60	92.60
	Maxout-CNN-BLSTM * [74]	87.60	-	-	-	-
	CNN + LSTM with view classifier * [23]	-	86.11	83.33	81.94	-
	CNN + LSTM without view classifier * [23]	-	86.67	85.00	82.22	-
2019	VGG-M + LSTM * [75]	91.38	-	-	-	91.38
2020	CNN(2D + 3D) without view classifier [76]	91.02	90.56	91.20	90.00	90.70
	CNN with view classifier [76]	91.02	90.74	92.04	90.00	90.95
2021	CNN without view classifier [77]	91.02	90.56	91.20	90.00	90.70
	CNN with view classifier * [77]	91.02	91.38	92.21	90.09	91.18

* Model trained with data augmentation.

**Table 4:** Comparison between the number of parameters and epoch times of the proposed method and different methods.

Model	Method	Number of Parameters	Epoch Time (s)			
			0°	30°	45°	60°
A	3D dense connection CNN + Bi-GRU + CTC	2,247,537	34.57	36.07	34.43	33.97
B	3D dense connection CNN + Multi-scale 3D CNN + Bi-GRU + CTC	3,456,369	36.48	36.58	34.93	35.43
C	3D dense connection CNN + Multi-scale 3D CNN + SAM + Bi-GRU + CTC	5,273,457	43.37	41.44	40.01	43.03
D	3D dense connection CNN + Multi-scale 3D CNN + SAM + RNN + CTC	2,429,362	35.27	36.78	36.15	35.86
E	3D dense connection CNN + Multi-scale 3D CNN + SAM + LSTM + CTC	3,905,458	38.16	39.13	38.94	37.45
F	3D dense connection CNN + Multi-scale 3D CNN + SAM + Bi-LSTM + CTC	6,421,426	54.35	53.18	53.04	57.86
G	3D dense connection CNN + Multi-scale 3D CNN + SAM + GRU + CTC	3,413,426	34.18	32.98	33.48	33.48
H	3D dense connection CNN + Multi-scale 3D CNN + SAM + Bi-GRU + Global self-attention + CTC	5,306,290	40.95	42.13	41.78	43.48
Our	3D dense connection CNN + Multi-scale 3D CNN + SAM + Bi-GRU + Local self-attention + CTC	5,306,290	40.46	41.39	41.97	42.41

## Data Availability

The database used in this article was OuluVS2. For details, please refer to [27].
